# Transient increase in CSF GAP-43 concentration after ischemic stroke

**DOI:** 10.1186/s12883-018-1210-5

**Published:** 2018-12-07

**Authors:** Åsa Sandelius, Nicholas C. Cullen, Åsa Källén, Lars Rosengren, Crister Jensen, Vesna Kostanjevecki, Manu Vandijck, Henrik Zetterberg, Kaj Blennow

**Affiliations:** 1 Institute of Neuroscience and Physiology, Department of Psychiatry and Neurochemistry, The Sahlgrenska Academy at the University of Gothenburg, Mölndal, Sweden; 2 Department of Neurology, Perelman School of Medicine, University of Pennsylvania, Philadelphia, USA; 3 Clinical Neurochemistry Laboratory, Sahlgrenska University Hospital, Mölndal, Sweden; 4 Institute of Neuroscience and Physiology, Department of Clinical Neuroscience and Rehabilitation, The Sahlgrenska Academy at University of Gothenburg, Gothenburg, Sweden; 5 Institute of Clinical Sciences, University of Gothenburg, Gothenburg, Sweden; 6 Fujirebio Europe nv, Ghent, Belgium; 7 UK Dementia Research Institute, WC1N, London, UK; 8 Department of Neurodegenerative Disease, UCL Institute of Neurology, Queen Square, London, UK; 9 Department of Psychiatry and Neurochemistry, Sahlgrenska University Hospital/Mölndal, S-431 80 Mölndal, Sweden

**Keywords:** GAP-43, Stroke, Neurodegeneration, Cerebrospinal fluid, Biomarkers

## Abstract

**Background:**

Cerebrospinal fluid (CSF) biomarkers reflect ongoing processes in the brain. Growth-associated protein 43 (GAP-43) is highly upregulated in brain tissue shortly after experimental ischemia suggesting the CSF GAP-43 concentration may be altered in ischemic brain disorders. CSF GAP-43 concentration is elevated in Alzheimer’s disease patients; however, patients suffering from stroke have not been studied previously.

**Methods:**

The concentration of GAP-43 was measured in longitudinal CSF samples from 28 stroke patients prospectively collected on days 0–1, 2–4, 7–9, 3 weeks, and 3–5 months after ischemia and cross-sectionally in 19 controls. The stroke patients were clinically evaluated using a stroke severity score system. The extent of the brain lesion, including injury size and degrees of white matter lesions and atrophy were evaluated by CT and magnetic resonance imaging.

**Results:**

Increased GAP-43 concentration was detected from day 7–9 to 3 weeks after stroke, compared to day 1–4 and to levels in the control group (*P* = 0.02 and *P* = 0.007). At 3–5 months after stroke GAP-43 returned to admission levels. The initial increase in GAP-43 during the nine first days was associated to stroke severity, the degree of white matter lesions and atrophy and correlated positively with infarct size (*r*_s_ = 0.65, *P* = 0.001).

**Conclusions:**

The transient increase of CSF GAP-43 is important to take into account when used as a biomarker for other neurodegenerative diseases such as Alzheimer’s disease. Furthermore, GAP-43 may be a marker of neuronal responses after stroke and additional studies confirming the potential of CSF GAP-43 to reflect severity and outcome of stroke in larger cohorts are warranted.

**Electronic supplementary material:**

The online version of this article (10.1186/s12883-018-1210-5) contains supplementary material, which is available to authorized users.

## Background

The presynaptic protein Growth associated protein 43 (GAP-43) is highly expressed during neuronal development and synaptogenesis and thereafter sustained in presynaptic terminals in the hippocampus and associate cortex in the adult human brain [1–3]. GAP-43 is involved in the regulation of axonal outgrowth, synaptic plasticity, and learning and memory functions [4–9]. Considering the function of GAP-43, it may also be induced by neuronal damage caused by stroke, traumatic brain injury and epilepsy [8, 10–14]. Highly elevated levels of GAP-43 protein in the peri-infarct region after experimentally induced cerebral ischemia have been well documented in rodents [11, 14–17]. Additionally, in rodent stroke models, a rapid induction of both GAP-43 gene and protein expression in the brain is reportedly present already at day one after ischemia and sustained up to 28 days and GAP-43 was suggested to be an early and sensitive marker of neuronal damage after ischemia [11, 17, 18]. However, GAP-43 quantification has to our knowledge not been measured in any body fluids of stroke patients. Novel stroke biomarkers could improve clinical diagnosis, predict prognosis, and guide therapeutic interventions. Furthermore, even though CSF GAP-43 concentration could become useful as a biomarker of neuronal injury after stroke, it is equally important to examine confounding conditions that may affect the use of CSF GAP-43 as a biomarker or outcome measurement in other neuronal diseases. We evaluated CSF GAP-43 concentration in longitudinally sampled stroke patients to 1) further determine the pathogenic mechanisms of increased CSF GAP-43 concentration, and 2) investigate the biomarker potential of CSF GAP-43 after stroke.

## Methods

### Study participants and sample collection

The study was approved by the ethics committee for medical research at the University of Gothenburg, Sweden. Longitudinal CSF samples were collected prospectively from 28 patients with an acute ischemic stroke that occurred within the first 3 days of admission at Sahlgrenska University Hospital, Department of Neurology, between September 1992 and January 1994. Exclusion criteria were patients with a history of previous stroke, malignant or autoimmune diseases, severe infections, or taking immunosuppressive drugs. When possible, CSF samples were collected on up to five occasions; at admission to the emergency ward at day 0–1, and then day 2–4, day 7–9, 3 weeks and 3–5 months. CSF samples were taken by lumbar puncture in the L3/L4 or L4/L5 interspace, the first 12 ml of CSF was collected in polypropylene tubes, centrifuged at 2000 g for 10 min, and stored at 80 °C pending biochemical analyses.

All stroke patients were clinically evaluated in a standardized way, as described previously [19]. Briefly, stroke severity was evaluated using a modified Scandinavian Stroke Scale Index (SSI) [19, 20]. The modified SSI is built on clinical scoring of seven parameters (between 1 and 5): consciousness, speech, facial paralysis, gait and physical strength in hand, arm and leg. In the modified version the maximum score is 27 and a higher score indicates increased severity of symptoms. Brain atrophy and infarct size was evaluated by magnetic resonance imaging (MRI) and computed tomography (CT) as previously described [19]. Atrophy in patients was scored as normal (*n* = 9), mild (*n* = 8), moderate (*n* = 4), and pronounced (*n* = 3), while white matter lesion status was scored as normal (*n* = 15), general-light (*n* = 1), general-moderate (*n* = 3), and cortical-light (*n* = 5) on T2-weighted MRI scans. Two patients lacked information about atrophy and white matter lesion, and two additional patients did not have enough samples for longitudinal analysis. Therefore, there were 24 patients for analysis of associations between these clinical measures and GAP-43 concentration. The control group consisted of healthy volunteers, without history, symptoms or signs of cognitive disturbances, neurological or psychiatric symptoms.

### Cerebrospinal fluid growth associated protein 43 enzyme-linked immunosorbent assay

CSF GAP-43 concentration was measured by an in house enzyme-linked immunosorbent assay (ELISA) described in detail previously [21], with minor modifications. Microwell modules (Thermo Fisher Scientific, Massachusetts, USA) were coated with a mouse anti-GAP-43 antibody (1.35 μg/ml NM4, Fujirebio, Ghent, Belgium) in carbonate buffer pH 9.6, overnight at 4 °C. After washing in 0.05%Tween/ phosphate buffered saline (PBST), wells were blocked with 2% non-fat milk/PBST (assay diluent) for 1 h at room temperature, frozen at − 20 °C until further use. After additional washes, in-house recombinant full length GAP-43 calibrators (78 pg/ml - 5000 pg/ml), blanks, control samples and CSF samples pre-diluted 1:2 in assay diluent were co-incubated with a rabbit detector antibody (0.20 μg/ml ABB-135, Nordic Biosite, Täby, Sweden) overnight at 4 °C. Then, plates were washed and incubated with anti-rabbit HRP (1:20000, IgG (H + L, Cross-adsorbed Secondary Antibody horse radish peroxidase, ThermoFisherScientific, USA) for 2 h in 1% bovine serum albumin/PBST. After subsequent washes, wells were incubated for 30 min with 3,3′,5,5′-tetramethylbenzidine (KemEnTech Diagnostics) in dark. The color reaction was stopped by addition of 0.2 M H_2_SO_4_ and the absorbance was read in a Sunrise microplate absorbance reader (Tecan group, Männedorf, Switzerland) at 450 nm (650 nm as reference value). Cerebrospinal fluid sample concentration was calculated via interpolation from the calibrator curve (4PL weighted 1/Y2).

### Assay validation

For CSF GAP-43 assay characterization, intra- and inter-assay precision, measurement range, recovery, parallelism, selectivity and sample storage stability was evaluated. For intra- and inter-assay precision, five duplicates of two samples with high and low concentrations, respectively, were analyzed on 5 different days. Two technicians were involved in the acquisition of data. For determination of measurement range (lower limit of quantification (LLOQ) and upper limit of quantification (ULOQ)), calibration curve data from 5 runs were used. The relative error of the back-calculated concentrations for the calibrators was plotted as a function of concentration, and LLOQ and ULOQ determined as calibrator points with < 20% relative errors. Parallelism was determined in duplicates of one sample analyzed as neat and diluted × 2, × 4, × 8, × 16 and × 32 times in assay diluent. Recovery was determined in two samples spiked with the GAP-43 calibrator. Neurogranin interference was determined in duplicates of one neat sample and the same with 10 ng/ml recombinant neurogranin protein spike was measured. Protein stability was evaluated by dividing 3 samples into nine aliquots where one was directly placed at − 80 °C, and the others stored at − 20 °C or at 4 °C overnight or for 1 week, at room temperature for 24 h, or freeze-thawed 1–4 times.

### Statistical analysis

Because the longitudinal trajectory of GAP-43 values appeared to be nonlinear, the data was initially analyzed in two parts. First, a linear mixed effects model was fit on measurements taken between before 9 days to test the hypothesis that GAP-43 increases directly after stroke. The model included random intercepts and slopes, and time was discretized into Days 0–1, Days 2–3, and Days 7–9. Age and sex were included as covariates and GAP-43 concentrations were normalized for each individual relative to their baseline measurement. Next, the same form of mixed model was fit on measurements taken from Days 7–9, 2–4 weeks, and 3–5 months in order to test the hypothesis that GAP-43 returns to normal levels over time. Differences in GAP-43 concentration across groups were analyzed using approximate *t* tests on the estimated marginal means. The Pearson correlation coefficient was calculated between subject-specific slopes for the first model of early measurements and the subject-specific slopes of the second model of late measurements to understand the relationship between initial change and late change in GAP-43 concentrations.

A linear mixed model was then fit on longitudinal stroke severity information, again with random intercepts and slopes. The relationship between GAP-43 change and stroke severity change was subsequently assessed by calculating the Pearson correlation coefficient between the subject-specific slopes of stroke severity and subject-specific slopes of GAP-43 as estimated from the respective mixed effects models. The association between GAP-43 change and infarct size was also computed using the Pearson correlation coefficient.

Additionally, group differences in GAP-43 change across discrete levels of white matter lesions and across discrete levels of brain atrophy were analyzed by fitting the same linear mixed effects model as above but with white matter lesion (or brain atrophy) status included as an interaction with time. Overall group difference was assessed using approximate *F* tests and between each pair of groups using approximate *t* tests on the estimated marginal means. Finally, concentrations of GAP-43 in healthy controls were compared to those of stroke patients at each time group using Mann-Whitney U tests, with correction for multiple comparisons using Holm’s procedure.

All tests were two-sided with a significance level set to *p* = 0.05. All statistical analysis was performed using the R programming language (version 3.4.3) with the *nlme* (version 3.1) and *emmeans* (version 1.2) packages being used for mixed effects modelling specifically.

## Results

### Patient demographics

Demographics of study participants are provided in Table 1. The control group was age matched to the clinical stroke group although with a larger percentage of females. There was no correlation between age and CSF GAP-43 concentration in stroke or control groups (*r*_s_ (day 0–4) = 0.43, *p* = 0.13; control: *r*_s_ = − 0.23, *p* = 0.34) and no differences between gender (stroke (day 0–4): *p* = 0.78; control: *p* = 0.45). For white matter lesion status in the stroke patients, there were nine individuals who scored as normal, eight as sporadic, four as moderate, and three as pronounced. For brain atrophy, 15 individuals were scored as normal, one as general-light, three as general-moderate, and five as cortical-light.

**Table 1:** Study participant demographics, clinical and biochemical data

	Controls	Stroke patients
Demographics		
No.	19	28
Age	66 (±7)	64 (±11)
Gender (% male)	47%	75%
SSI 0 < 15 < 30 (n)		23/ 5/ 0
White matter lesion: normal/sporadic/moderate/ pronounced (n)		9/ 8/ 4/ 3
Atrophy: normal/ general sporadic/general moderate/cortical sporadic (n)		15/ 1/ 3/ 5
GAP-43 (pg/ml)	2711 (2021–3602)	
Day 0–1 (*n* = 8)		2008 (1762–2954)
Day2–4 (*n* = 23)		3311 (2057–5491)
Day7–9 (*n* = 26)		3819 (2531–7242)
3 weeks (*n* = 22)		3949 (2668–6606)
3–5 months (*n* = 24)		2562 (2112–2821)

### Assay validation results

The average intra- and inter-assay variation was assessed by determining the coefficient of variation (CV) of repeated analysis. The intra- and inter-assay CVs were 3.4 and 7.4% respectively, for a sample with a mean GAP-43 concentration of 1184 pg/ml, and 3.2 and 5.8% respectively, for a sample with a mean GAP-43 concentration of 3117 pg/ml. The LLOQ was 315 pg/ml and ULOQ was 10,000 pg/ml. The parallelism was 85–100% for samples diluted 2–8 times. Recovery was 99–109%. No neurogranin interference was detected (spike sample (1751 pg/ml); GAP-43 recovery was 101% of neat sample (1734 pg/ml)). All stability aliquots were analyzed simultaneously and their concentrations differed between 81 and 116% for samples with different storage conditions, and 75–115% for freeze-thaw cycled aliquots, compared to the original sample stored at − 80 °C.

### CSF GAP-43 concentration increases directly after stroke and eventually returns to initial levels

**Fig. 1:**
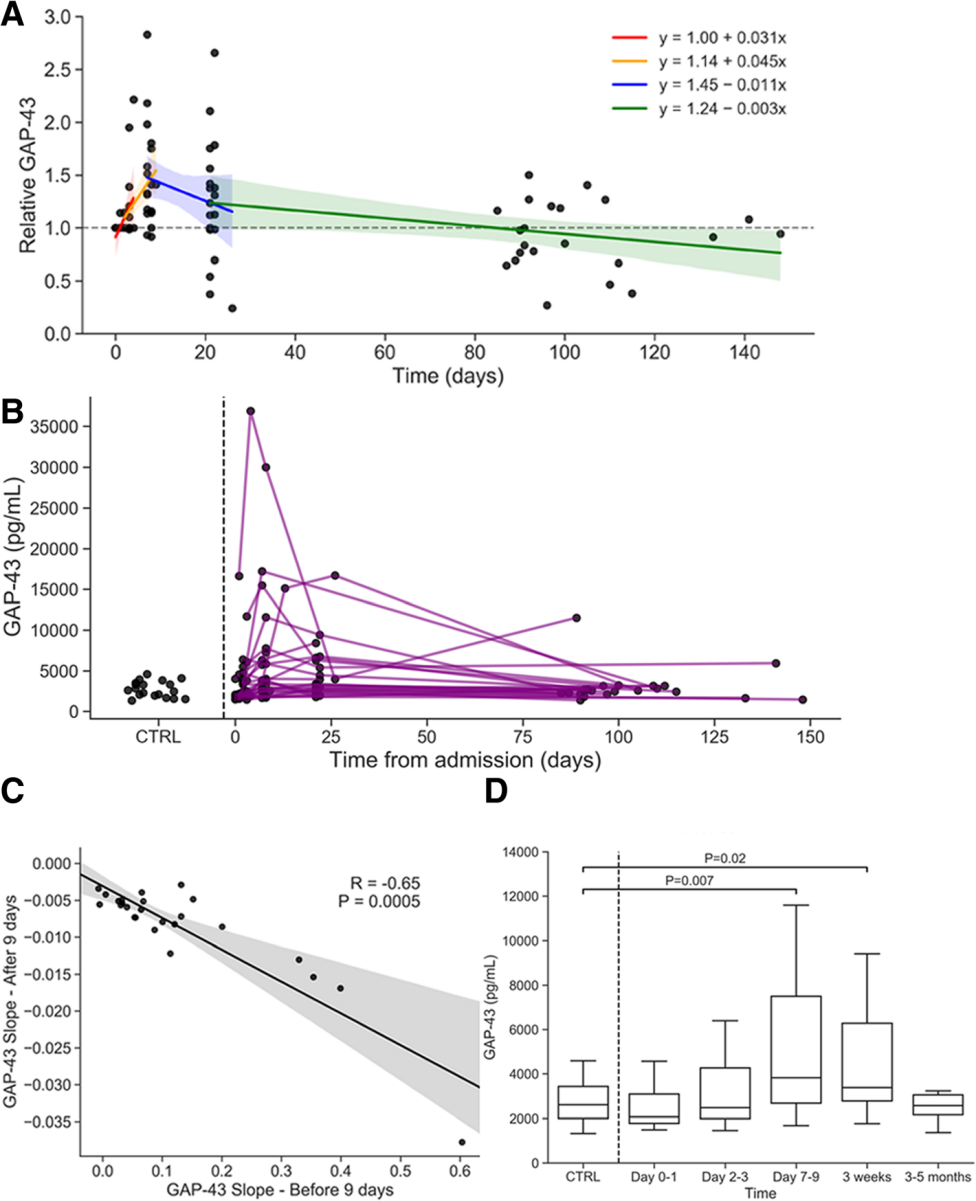
Transient increase in CSF GAP-43 concentration after stroke. **a** GAP-43 concentration relative to each individual’s baseline and a piecewise-linear regression model displaying overall longitudinal trends between the five time points; **b** Baseline GAP-43 levels in controls (left of dotted line) and continuous individual longitudinal trajectories of absolute GAP-43 concentration after stroke, where each line represents a single individual (right of dotted line), showed a transient increase directly after stroke which returned to control levels for most individuals; **c** Relationship between the increase in GAP-43 concentration before 9 days and the subsequent decrease in GAP-43 concentration after 9 days; **d** GAP-43 concentration (median and interquartile range) in controls (left of dotted line) and in stroke patients at Day 0–1, Day 2–3, Day 7–9, 3 weeks and 3–5 months

CSF GAP-43 concentrations in controls and change over time in stroke patients are depicted in Fig. 1. GAP-43 concentration increased significantly over time relative to baseline in the first 9 days after stroke (relative change: + 19% per day, 95% CI [11.1, 26.9]; F_1,32_ = 24.8, *P* < 0.0001, Fig. 1a). This result also held when viewing GAP-43 in absolute terms (absolute change: + 506.6 pg/mL per day, 95% CI [204.0, 809.3], F_1,32_ = 11.7, *P* = 0.002, Fig. 1b). Additionally, GAP-43 concentrations between Days 7–9 were significantly elevated compared to Days 0–1 (relative difference: 86.4, 95% CI [11.8, 161.0]; t_31_ = 2.4, *P* = 0.02, Fig. 1a) and compared to Days 2–3 (relative difference: 74.5, 95% CI [15.8, 133.4]; t_31_ = 2.6, *P* = 0.02, Fig. 1a).

Additionally, GAP-43 concentration decreased significantly starting 9 days after stroke relative to baseline (relative change: − 0.6% per day, 95% CI [0.3, 1.2]; F_1,38_ = 4.5, *P* = 0.04) and absolute GAP-43 concentrations also decreased significantly over the same period (absolute change: − 30.2 pg/mL per day, 95% CI [− 5.7, − 54.7]; F_1,38_ = 6.2, *P* = 0.02). Additionally, GAP-43 concentrations between 3 and 5 months were significantly lower compared to 3 weeks (relative difference: 86.1, 95% CI [2.1, 170.2]; t_40_ = 2.6, *P* = 0.04) and compared to Days 7–9 (relative difference: 81.3, 95% CI [− 4.2, 166.8]; t_40_ = 2.4, *P* = 0.04).

Overall, GAP-43 concentration increased slightly relative to baseline (mean: 100%) in Days 2–3 (mean: 118.1%) and increased greatly in Days 7–9 (mean: 187.1%), then began to decrease relative to Days 7–9 after 3 weeks (mean: 166.6%) and returned to initial levels after 3–5 months (mean: 89.8%). Continuous individual longitudinal trajectories of absolute GAP-43 concentration after stroke are shown in Fig. 1b, where each line represents a single individual (right of dotted line), showing that most stroke suffering individuals returned to control levels (Fig. 1b). The initial increase in GAP-43 concentration before 9 days was highly correlated with the subsequent decrease in GAP-43 levels over time (*r* = − 0.65, *P* = 0.0005; see Fig. 1c), indicating that the individuals with the largest initial increases in GAP-43 were more likely to have larger subsequent decreases in GAP-43 concentration later on.

Compared to cross-sectional measurements of healthy controls, absolute GAP-43 concentration in stroke patients was significantly elevated in Days 7–9 (median difference: 1628 pg/mL, 95% CI [333, 3905]; *P* = 0.007, Fig. 1d) and 3 weeks after stroke (median difference: 1115 pg/mL, 95% CI [106, 2467]; *P* = 0.02, Fig. 1d), but not in Days 0–1 (median difference: − 207 pg/mL, 95% CI [− 1252, 537]; *P* = 0.58, Fig. 1d), Days 2–3 (median difference: 270 pg/mL, 95% CI [− 390, 1388]; *P* = 0.44, Fig. 1d), or 3–5 months (median difference: − 128 pg/mL, 95% CI [− 774, 567]; *P* = 0.73, Fig. 1d) after stroke.

### Association between GAP-43 change and stroke severity, white matter lesions, brain atrophy and infarct size

**Fig. 2:**
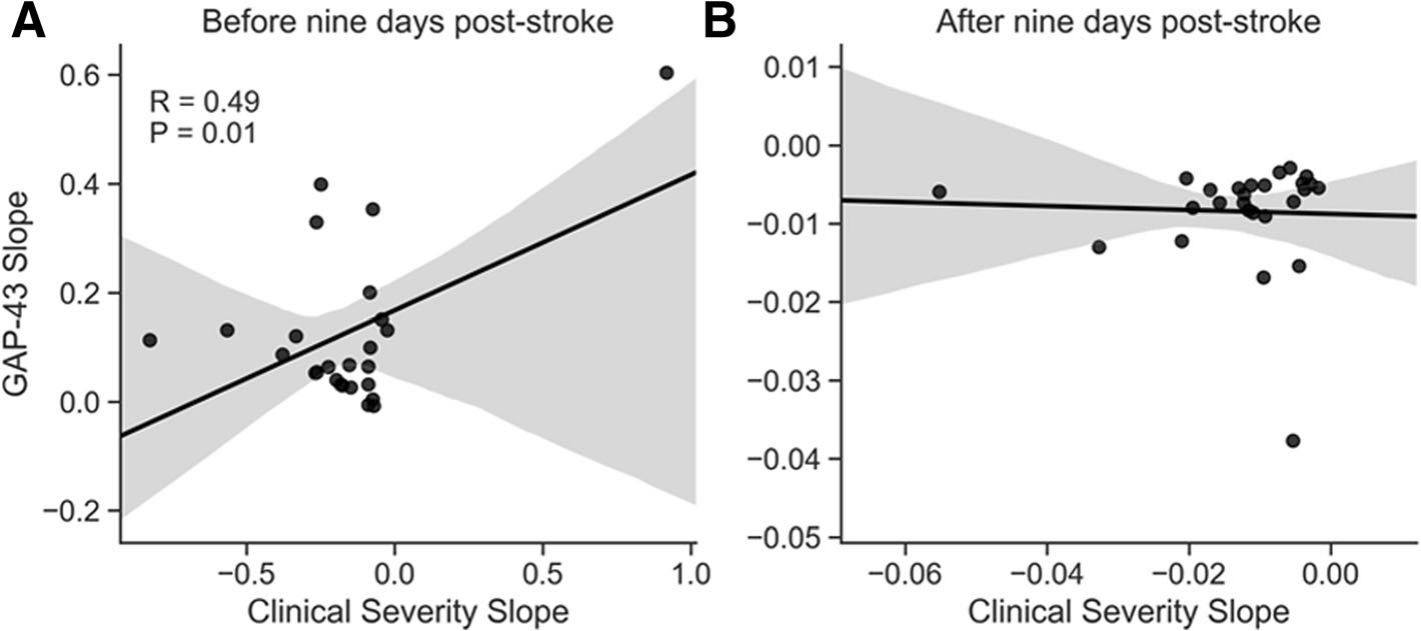
Association of CSF GAP-43 to clinical characteristics. **a** Relationship between increase in GAP-43 concentration and the worsening of clinical stroke severity score in the period until 9 days after stroke; **b** the same as panel a, but for the period after 9 days after stroke

In the first 9 days after stroke, the increase in GAP-43 concentration was significantly associated with change in stroke severity as measured by the modified SSI scale (*r* = 0.49, *P* = 0.01, Fig. 2a). This association was strengthened when considering only those patients whose SSI scores improved over time (*r* = 0.82, *P* = 0.002). However, there was no correlation between the change in GAP-43 concentration over time and the change of stroke severity (*r* = − 0.05, *P* = 0.84, Fig. 2b) in the time period after 9 days.

The change in GAP-43 concentration until 9 days after stroke significantly differed overall across white matter lesion status (F_3,27_ = 4.1, *P* = 0.017), but not for change in GAP-43 concentration later on (F_3,33_ = 0.61, *P* = 0.61). However, there was no significant difference between GAP-43 changes for any two specific white matter lesion groups likely due to lack of statistical power (see Additional file 1 for F and *P*-values).

Additionally, the change in GAP-43 concentrations up to 9 days after stroke again significantly differed across brain atrophy status (F_3,26_ = 6.1, *P* = 0.003), but not for GAP-43 concentrations after 9 days (F_3,31_ = 0.14, *P* = 0.93). Again, there was no significant difference between GAP-43 changes for any two specific brain atrophy groups likely due to lack of statistical power (see Additional file 1 for F and P-values).

The initial increase in GAP-43 concentration over the first 9 days correlated positively with infarct size (*r* = 0.75, *P* < 0.0001), indicating that those with higher infarct size also had larger initial increase in GAP-43 levels. Meanwhile, the later decrease in GAP-43 concentration after 9 days correlated negatively with infarct size (*r* = − 0.64, *P* = 0.001).

## Discussion

In this study, we detected a transient increase in CSF GAP-43 concentration after acute ischemic stroke that was most prominent during the first 2 weeks after injury. As CSF is in direct contact with the brain, its content can reflect ongoing processes in the brain tissue. Animal models of ischemia suggest that the content of GAP-43 in the brain may be the result of both an increase due to regeneration responses and a decrease due to disrupted axons at the site of injury [11, 17, 18]. The temporary increase in CSF GAP-43 in our study likely reflects an increase due to regenerative responses, however additive effects of leakage of this synaptic protein from damaged neurons cannot be ruled out. Substantial axonal sprouting and cortical reorganization is initiated after stroke and reported in patients and experimental stroke models [22–26].

The CSF GAP-43 concentration in control subjects was similar to most stroke patients at admission (Day 0–1, Day 2–3) and the following increase during the first 9 days after stroke indicate that increased CSF GAP-43 results from a delayed or progressing process. Different mechanisms of ischemia-induced cell death are acutely activated in the ischemic core, appearing within a time-window of 0–2 days [27]. In addition, delayed neuronal death is known to appear several days after ischemia and progressing weeks after the injury [28, 29]. The delayed increase of GAP-43 in CSF may reflect the time it takes for neuronal injury to spread across larger peri-infarct areas, or reflect the time it takes for GAP-43 expressing neurons to die, with release of GAP-43 to the extracellular space and CSF. This is further supported by a study reporting decreased numbers of GAP-43 expressing cortical cells in post-ischemic patients [30]. Also, GAP-43 expressing neurons may be more resistant to the direct ischemic event. It has been suggested that different types of neurons are differently susceptible to early and delayed cell death after ischemic injuries [31].

Moreover, our results suggest an association of CSF GAP-43 concentration and clinical characteristics during the first 2 weeks after ischemia. Larger increase in CSF GAP-43 was related not necessarily to a worsening in clinical characteristics but rather a lack of improvement, given that all but one individual either improved or stayed the same over the course of the study. This connection was even stronger when analyzing only those patients whose clinical characteristics actually improved. The connection to white matter lesions indicates that GAP-43 levels were likely to increase more in individuals with pronounced white matter lesions. Similarly, a high increase in CSF GAP-43 concentration was related to more severe atrophy and larger infarct size. Together with weaker, or the lack of, association with clinical measures at later time points, this suggests that CSF GAP-43 concentration primarily reflect the degree of, or response to, neuronal injury but not recovery. Limitations of this study include the relatively low sample number and the rather long sample storage time. The reason for the long sample storage time is that a stable CSF GAP-43 quantification method was just recently developed and this evaluation of CSF GAP-43 in longitudinal stroke samples was possible through a previous established collaboration. However, longitudinal studies have the advantage of letting each patient be its own control at baseline and individual changes over time should not be considerably affected assuming equal effects of storage on all samples. An additional limitation is the use of the modified version of the SSI, which is not standardized internationally. Nonetheless, we show for the first time that changes in CSF GAP-43 after stroke are connected to clinical measures. Additional studies are required to further evaluate the value of GAP-43 as a stroke biomarker and elucidate its connections to injury progression and clinical outcome. CSF GAP-43 could become useful as a quantitative measure of the regenerative response during recovery, which should simplify comparison and evaluation of both stroke treatments and rehabilitation strategies.

Importantly, the results reported here, demonstrating a clear effect of ischemia on CSF GAP-43 concentrations, need to be considered when using CSF GAP-43 as a biomarker in other settings. In a previous study of several dementia disorders, we have shown a specific increase in CSF GAP-43 in Alzheimer’s disease compared to several other neurodegenerative diseases [21]. As increased CSF GAP-43 concentration was associated to Alzheimer’s disease neuropathology and correlated with cognitive decline, it could be useful as an outcome marker in clinical trials for novel Alzheimer’s disease therapeutics. As stroke is likely to occur in a fraction of the study population during a drug trial period, it would be important to take into consideration that CSF GAP-43 is transiently increased after stroke affecting outcome levels. Other CSF biomarkers such as t-tau, p-tau and neurogranin have also been evaluated in stroke as well as traumatic brain injury to elucidate mechanisms of neuronal injury reflected by particular biomarkers [32–36]. It was found that t-tau, but not p-tau, increased after stroke suggesting they reflect different disease/injury mechanisms [33, 36]. Similar to our findings, CSF t-tau and neurogranin concentrations were related to stroke characteristics [32, 34]. Although CSF GAP-43 was previously found largely selective for Alzheimer’s disease among neurodegenerative diseases, it may be altered by yet other conditions besides stroke. Changes of GAP-43 expression levels in experimental models of epilepsy and traumatic brain injury urges for additional investigations of CSF GAP-43 change in epilepsy and traumatic brain injury patients to find out the potential use of GAP-43 concentration as a neuronal injury biomarker.

## Conclusion

The transient increase of CSF GAP-43 appearing most intensely at 1–3 weeks after stroke is important to take into account when used as a biomarker for other neurodegenerative diseases. Our findings also suggest that CSF GAP-43 may be a marker of neuronal injury responses in stroke and urge for additional studies confirming the potential of CSF GAP-43 to reflect severity and outcome of stroke in larger cohorts.

## Additional file

Additional file 1: Table of t and *p* values across the different white matter lesion status and brain atrophy status groups, up to 9 days and, after 9 days past the stroke. (XLSX 9 kb)

## Abbreviations

CSF: Cerebrospinal fluid
CT: Computed tomography
ELISA: Enzyme-linked immunosorbent assay
GAP-43: Growth associated protein 43
LLOQ: Lower limit of quantification
MRI: Magnetic resonance imaging
SSI: Scandinavian Stroke Scale Index
ULOQ: Upper limit of quantification
